# Enoxacin Elevates MicroRNA Levels in Rat Frontal Cortex and Prevents Learned Helplessness

**DOI:** 10.3389/fpsyt.2014.00006

**Published:** 2014-02-10

**Authors:** Neil R. Smalheiser, Hui Zhang, Yogesh Dwivedi

**Affiliations:** ^1^Department of Psychiatry, Psychiatric Institute, University of Illinois at Chicago, Chicago, IL, USA; ^2^Department of Psychiatry and Behavioral Neurobiology, University of Alabama at Birmingham, Birmingham, AL, USA

**Keywords:** miRNAs, depression, enoxacin, behavior, rat

## Abstract

Major depressive disorder (MDD) is a major public health concern. Despite tremendous advancement, the pathogenic mechanisms associated with MDD are still unclear. Moreover, a significant number of MDD subjects do not respond to the currently available medication. MicroRNAs (miRNAs) are a class of small non-coding RNAs that control gene expression by modulating translation, mRNA degradation or stability of mRNA targets. The role of miRNAs in disease pathophysiology is emerging rapidly. Recently, we reported that miRNA expression is down-regulated in frontal cortex of depressed suicide subjects, and that rats exposed to repeated inescapable shock show differential miRNA changes depending on whether they exhibited normal adaptive responses or learned helpless (LH) behavior. Enoxacin, a fluoroquinolone used clinically as an anti-bacterial compound, enhances the production of miRNAs *in vitro* and in peripheral tissues *in vivo*, but has not yet been tested as an experimental tool to study the relation of miRNA expression to neural functions or behavior. Treatment of rats with 10 or 25 mg/kg enoxacin for 1 week increased the expression of miRNAs in frontal cortex and decreased the proportion of rats exhibiting LH behavior following inescapable shock. Further studies are warranted to learn whether enoxacin may ameliorate depressive behavior in other rodent paradigms and in human clinical situations, and if so whether its mechanism is due to upregulation of miRNAs.

## Introduction

Major depressive disorder (MDD) is one of the most prevalent psychiatric disorders. It affects about 17% of Americans during their lifetime (1) and is associated with psychosocial impairment, poor quality of life, significant disability (2), morbidity, and mortality (3–5). MDD is being diagnosed at early ages, and about 25% of people diagnosed with MDD are below 19 years. Although much work has been done to characterize MDD, about 40% of MDD patients do not respond to the currently available medications (6). This is partially a result of poor understanding of the molecular pathophysiology underlying MDD.

Compromised neural and structural plasticity has consistently been associated with MDD (7). The cellular mechanisms that underlie such compromised neural plasticity and structural impairments in MDD are not clearly understood and no single mechanism appears to be responsible for MDD etiopathogenesis; however, it is becoming increasingly evident that MDD may result from disruptions across whole cellular networks, leading to aberrant information processing in the circuits that regulate mood, cognition, and neurovegetative functions (7). In fact, evidence demonstrating impaired cellular networks that regulate neural plasticity has reshaped our views about the neurobiological underpinnings of MDD (8).

In recent years, the emergence of small non-coding RNAs as coordinated regulators of gene expression that target families of RNA sequences has gained much attention in neuropsychiatric disease pathophysiology (9). These small non-coding RNAs regulate gene expression by several mechanisms including ribosomal RNA modifications, repression of mRNA expression by RNA interference, alternative splicing, and regulatory mechanisms mediated by RNA–RNA interactions. Small non-coding RNAs include microRNAs (miRNAs), small nucleolar RNAs, small interfering RNAs, piwi-interacting RNAs, spliceosomal RNAs, and p/MRP genes. Among them, miRNAs are the most studied and well characterized and have emerged as major regulators of neural plasticity and higher brain functioning (10).

The relation between miRNA expression and depressive behavior is not straightforward. On the one hand, we recently reported that there is a global down-regulation of miRNA expression in prefrontal cortex of depressed suicide subjects (11), and have replicated this finding in an additional cohort (12). On the other hand, when rats were exposed to repeated inescapable shock, those which adapted normally [non-learned helpless (NLH) rats] showed strong down-regulation of a specific miRNA module whereas those which exhibited learned helpless behavior (LH rats) had a blunted miRNA response (13).

In order to investigate further the relation between miRNA expression and any type of behavior, it is desirable to have an experimental tool to manipulate miRNA levels directly and independently. Enoxacin is a fluoroquinolone antibiotic that (among other actions) binds HIV-1 TAR RNA binding protein (TRBP), stabilizing the dicer–TRBP complex and raising miRNA levels globally (14–16). Although enoxacin passes the blood–brain barrier sufficiently to exert anti-bacterial effects in meningitis (17), it is not known whether enoxacin, administered peripherally in usual therapeutic doses, will have effects on miRNA levels in brain. Thus, the present study aims to examine whether enoxacin increases expression of selected miRNAs, which are enriched in brain and are involved in synaptic plasticity and neurogenesis (9). We also tested whether enoxacin is able to produce anti-depressant effects in a standard rodent paradigm, learned helplessness following inescapable shock (18).

## Materials and Methods

### Animals

Virus-free male Holtzman (Sprague-Dawley) rats (Harlan Laboratories, Inc., Indianapolis, IN, USA) were housed in individual cages under standard laboratory conditions (temperature 21 ± 1°C, humidity 55 ± 5%, 12-h light/dark cycle). Animals were provided free access to food and water. Animals were housed for 3 weeks before the experiment, and the body weight was 325–350 g (11–12 weeks of age) at the start of the experiment. All the experiments were performed between 8 and –10 a.m.

### Enoxacin treatment

#### Experiment 1

**Figure 1:**
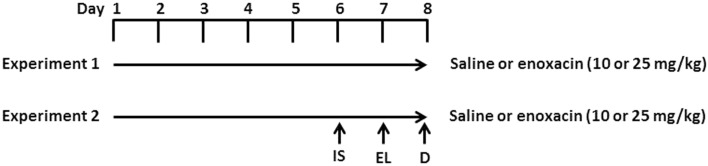
**Experimental design**. In experiment 1, rats were given i.p. injections of enoxacin (10 or 25 mg/kg) or saline for 8 days. One hour after the last enoxacin injection on day 8, rats were decapitated (D). In experiment 2, rats were given i.p. injections of enoxacin (10 or 25 mg/kg) or saline for 8 days. On the sixth day, rats were given inescapable shock (IS) and tested for escape latency (EL) on day 7. One hour after the last enoxacin injection on day 8, rats were decapitated.

Enoxacin treatment is described in Figure 1. Rats were given saline, 10 or 25 mg/kg enoxacin (EMD Millipore Corporation, Billerica, MA, USA catalog no. 557305) intraperitoneally (i.p.), once daily for 8 days (*n* = 12–14 in each group). Rats were decapitated 1 h after the last dose (Figure 1).

#### Experiment 2

To test whether enoxacin prevents depressive behavior, 10 rats were treated with saline, 8 rats were treated with 10 mg/kg of enoxacin, and 8 rats were treated with 25 mg/kg enoxacin for 6 days prior to subjecting animals to inescapable shock (day 6) and assessing for learned helplessness in the escape latency test (day 7). Animals were treated with enoxacin 1 h prior to inescapable shock (day 6) and escape latency test (day 7). The enoxacin treatment continued on days 7 and 8 (Figure 1). Induction of LH behavior is described in our earlier publication (18). Briefly, rats were placed in Plexiglass tubes and shocks were delivered by means of a computer-controlled constant current shock generator to electrodes augmented with electrode paste to the rat’s tail. The inescapable shock consisted of 100 random shocks delivered for 5 s at the rate of 1.0 mA, with a mean interval of 60 s. Another sham group (four treated with saline, four treated with enoxacin 10 mg/kg, and four treated with enoxacin 25 mg/kg) was placed in Plexiglass tubes but was not subjected to shocks. The depressive behavior was tested in a shuttle box as described earlier (18). Footshock was delivered through the grid floor by a shock generator. The shuttle escape testing began with five trials (FR-1) during which a single crossing would terminate the shocks. This was followed by 25 trials (FR-2) in which a rat had to cross from one side of the shuttle box to the other and come back to terminate the shocks. Shocks were terminated automatically after 30 s if there was no response within that time. The intensity of the shocks was 0.6 mA. The shocks were presented on a variable schedule. There was a 5-min interval between FR-1 and FR-2. Shuttle escape latencies were recorded automatically by a computer attached to the generator and shuttle box.

Rats were divided into two groups based on the mean latency observed after FR-2: (1) those rats in which the mean latency was ≥20 s (termed LH) and (2) those in which the mean latency was <20 s (termed as NLH). The mean latency ≥20 s cut-off was chosen based on the previous studies showing that this escape latency is reliable in determining the LH behavior (19). In our study, we found that about 50–60% of rats showed LH behavior. This is consistent with our previous studies (9, 13, 20–23). The rats that were confined to Plexiglass tubes and were not shocked were also tested and termed as sham rats. Rats were decapitated 24 h after the last escape testing.

### miRNA expression

Total RNA was isolated in samples of frontal cortex using a modified protocol designed to optimize recovery of small RNAs (11, 24). Total RNA was isolated with Trizol reagent (Invitrogen Life Technologies, USA) according to the manufacturer’s directions. GlycoBlue 20 μg (Ambion) was added to the RNA precipitation step, which was allowed to proceed overnight at −20°C. The RNA pellet was centrifuged at 20,000 × *g* for 25 min at 4°C, rinsed with 80% ethanol in DEPC-treated water (Invitrogen Life Technologies, Carlsbad, CA, USA), resuspended and treated with RNAsecure (Ambion, Grand Island, NY, USA), and treated with DNase I using DNA-free TURBO kit (Ambion, Grand Island, NY, USA). RNA was treated with DNAse I and checked for purity by OD 260:280 ratio (NanoDrop 1000 Spectrophotometer, Thermo Scientific, Wilmington, DE, USA).

Expression levels of four selected miRNAs (mir-124, mir-125a, mir-132, and let-7a) were measured in the frontal cortex by real-time PCR using TaqMan primers and probes as described earlier (11, 24).

Briefly, 1 μg of total RNA was reverse transcribed using 50 ng random hexamers, 2 mM dNTP mix, 10 μm ribonuclease inhibitor, and 200 μm MMLV-reverse transcriptase enzyme in a final reaction volume of 20 μl. The primer/probe sets for all target genes and endogenous controls were obtained from Applied Biosystems (Foster City, CA, USA) as the TaqMan Gene Expression Assay kit. To determine the linear range and sensitivity of the kits, a standard curve was generated using serial 10-fold dilutions. Only PCR reactions showing efficiencies above 95% were considered acceptable. All miRNAs tested had efficiencies similar to the endogenous controls and were run in parallel with the endogenous controls. The PCR reaction was carried out in a final volume of 20 μl, containing 5 μl of cDNA diluted 1:10 with DEPC water, 1× of TaqMan primer/probe mix (20×), and 1× TaqMan^®^ Universal PCR Master Mix (Applied Biosystems, Foster City, CA, USA). For each primer/probe tested, the PCR reaction also included a non-reverse transcription negative control to confirm the absence of genomic DNA, and a non-template negative control to check for primer–dimer. All experiments were performed in duplicate as follows: denaturation at 95°C for 10 min followed by 40 cycles of a two-step program [denaturation at 95°C for 15 s and annealing/extension at 60°C for 1 min on the Mx3005p (Agilent Technologies, Santa Clara, CA, USA). All samples were run on a 2% agarose gel to confirm specificity. The amounts of target genes expressed were normalized to GAPDH and showed no significant variation in our sample set, Table 2]. Fold changes between groups were measured using the 2^−ΔΔCt^ method, where ΔΔ*C*_T_ = (*C*_T target_ − *C*_T normalizer_)_sample_ − (*C*_T target_ − *C*_T endogenous gene_)_control_.

## Results

As shown in Table 1, enoxacin (10 mg/kg) increased miRNA abundance levels of the selected miRNAs by 3- to 12-fold, and 25 mg/kg pretreatment increased miRNA levels by 4- to 22-fold above the levels observed in saline-treated rats. This demonstrates that “usual” therapeutic doses of enoxacin are quite effective in raising miRNA levels in the cortical area of the brain in a dose-dependent manner.

**Table 1:** **miRNAs expression in rat frontal cortex after saline or enoxacin treatment**.

Group	Let-7a		miR-124		miR-125a-5p		miR-132		GAPDH mRNA	
	Ct^a^	Fold-change compared to sham^b^	Ct	Fold-change compared to sham	Ct	Fold-change compared to sham	Ct	Fold-change compared to sham	Ct	Fold-change compared to sham
Saline-treated *N* = 14	21.98 ± 0.52	1.00	21.53 ± 0.45	1.00	22.61 ± 0.84	1.00	20.94 ± 0.44	1.00	18.51 ± 0.39	1.00
Enoxacin (10 mg/kg) *N* = 12	18.39 ± 1.74	12.34	19.54 ± 1.5	4.08	19.28 ± 2.05	10.38	19.43 ± 1.06	2.92	18.55 ± 0.42	0.97
		(*p* = 6.9 × 10^−8^)		(*p* = 0.000038)		(*p* = 0.0000048)		(*p* = 0.000046)
Enoxacin (25 mg/kg) *N* = 12	17.84 ± 0.91	17.90	18.99 ± 2.68	5.93	18.1 ± 1.09	22.33	18.81 ± 0.71	4.46	18.54 ± 0.34	0.98
		(*p* = 1.2 × 10^−13^)		(*p* = 0.00091)		(*p* = 8.9 × 10^−12^)		(*p* = 0.00002)

^a^ Indicated are means ± SD of Ct values in each group. Note that Ct values follow a log_2_ scale (i.e., a difference in Ct values of 1 means a twofold change in abundance);

*^b^ sham (no shock) groups were treated with saline or with enoxacin 10 or 25 mg/kg for 1 week; *t*-test *p* values are in parentheses*.

When rats were pretreated with enoxacin and subjected to inescapable shock, the LH phenotype was suppressed at both doses (Table 2). Mean escape latency for the saline group was 19.16 s, whereas for the 10 mg/kg group, it was 9.85 s, and for 25 mg/kg, it was 7.73 s. Non-parametric statistics (two-tailed Mann–Whitney *U* test) showed statistically significant differences between groups: saline vs. 10 mg/kg, *p* = 0.0409; saline vs. 25 mg/kg, *p* = 0.0505; and saline vs. combined enoxacin group, *p* = 0.0177.

**Table 2:** **Escape latency of individual rats when given no shock or inescapable shock and simultaneously treated with saline or enoxacin (10 or 25 mg/kg)**.

	Treatment	Escape latency (s)	Response to shock	Enoxacin (mg/kg)	Escape latency (s)	Response to shock	Enoxacin (mg/kg)	Escape latency (s)	Response to shock
Sham (no shock)	Saline	3.27		10	2.76		25	4.22	
Sham (no shock)	Saline	4.41		10	3.15		25	3.29	
Sham (no shock)	Saline	4.63		10	5.22		25	4.37	
Sham (no shock)	Saline	2.68		10	3.17		25	5.82	
Shock	Saline	26.13	LH	10	21.36	LH	25	17.19	NLH
Shock	Saline	26.53	LH	10	23.78	LH	25	3.91	NLH
Shock	Saline	27.15	LH	10	8.62	NLH	25	8.66	NLH
Shock	Saline	30.46	LH	10	4.47	NLH	25	2.83	NLH
Shock	Saline	28.22	LH	10	1.68	NLH	25	3.96	NLH
Shock	Saline	27.34	LH	10	12.27	NLH	25	5.32	NLH
Shock	Saline	11.9	NLH	10	2.78	NLH	25	7.14	NLH
Shock	Saline	3.9	NLH	10	3.82	NLH	25	12.86	NLH
Shock	Saline	6.18	NLH						
Shock	Saline	4.19	NLH						

*For rats exposed to shock, escape latency <20 s = non-learned helpless (NLH); ≥20 s = learned helpless (LH). As a baseline control, sham rats were given no shock on day 6 but were tested for escape latency on day 7*.

We repeated the enoxacin effects on behavioral outcome for a second time. The rats were treated with enoxacin (10 or 25 mg/kg doses) for 8 days as discussed in experiment 2. A total of 26 rats were examined (*n* = 8 for sham saline-treated rats, *n* = 9 for 10 mg/kg enoxacin, and *n* = 9 for 25 mg/kg enoxacin). We found that enoxacin produced similar results as in Table 1 such that only two animals out of nine showed LH behavior at 10 mg/kg enoxacin and two animals out of nine showed LH behavior at 25 mg/kg (Table A1 in Appendix).

## Discussion

Enoxacin belongs to a family of synthetic anti-bacterial compounds, the fluoroquinolones, which function as bacterial type II topoisomerase inhibitors (25). Shan et al. (14) showed that enoxacin and some of its analogs promote the biogenesis of endogenous miRNAs in mammalian cells by binding to TRBP, stabilizing the complex between dicer and TRBP, and enhancing dicer-mediated precursor processing and/or loading onto RNA silencing complex (RISCs). This is further confirmed by a recent study, which shows that enoxacin enhances the production of miRNAs with tumor suppressor functions by binding to the miRNA biosynthesis protein TRBP2 (15).

In the present study, we examined the effect of enoxacin on the expression of select miRNAs. These include: let-7a, miR-124, miR-125a-5p, and miR-132. Although we expect that enoxacin will increase the expression of miRNAs globally, these miRNAs were chosen because of their importance in neuronal cell biology. miR-124 is involved in neurogenesis and is associated with the differentiation status of neuronal cells in mouse brain (26). By targeting glypican-4, miR-125 regulates cell growth (27). MicroRNA-125 also promotes neuronal differentiation in human cells by repressing multiple targets (28) and in mammalian neurons, miR-125 is associated with regulation of dendritic spine length (29). Let-7 is involved in neurogenesis (30) as well as neuronal development and function (31). BDNF regulates protein synthesis via let-7. BDNF stimulation upregulates Lin28, an RNA binding protein that can bind precursors of let-7, preventing them from being processed by the Dicer–TRBP machinery. The resulting diminished levels of mature let-7 miRNAs relieve repression of mRNAs with let-7 binding sites and permit their translation (32). In addition, let-7 regulates dendritic spine density along the length of neurons (33). Expression of miR-132 enhances neurite outgrowth, dendritic morphogenesis, and spine formation (34–37), and is induced by BDNF via CREB. It has been shown that CREB- and activity-regulated miR-132 is necessary and sufficient for hippocampal spine formation. Expression of the miR-132 target, p250GAP, is inversely correlated with miR-132 levels and spinogenesis. Furthermore, knockdown of p250GAP increases spine formation while introduction of a p250GAP mutant unresponsive to miR-132 attenuates this activity. Inhibition of miR-132 decreases both mEPSC frequency and the number of GluR1-positive spines, while knockdown of p250GAP has the opposite effect. Additionally, miR-132/p250GAP circuit regulates Rac1 activity and spine formation by modulating synapse-specific Kalirin7–Rac1 signaling. These results suggest that neuronal activity regulates spine formation, in part, by increasing miR-132 transcription, which in turn activates a Rac1–Pak actin remodeling pathway. All of these miRNAs are processed by dicer and, in other systems, have been shown to respond to enoxacin (14–16).

To our knowledge, the present report is the first report to show that enoxacin increases expression of miRNAs in brain and that enoxacin affects behavioral responses of any kind. Furthermore, enoxacin given at doses within the usual anti-bacterial therapeutic range suppressed learned helplessness in rats, a standard model of depressive behavior. Further work is needed to learn the exact mechanism by which enoxacin prevented LH behavior, and whether lower doses of enoxacin that produce more modest changes in miRNA expression will also be behaviorally significant. It will also be interesting to test enoxacin in other types of behavior and in other models of depression and post-traumatic disorder. For example, using a repeated LH paradigm that produces a prolonged depressive phenotype (18), one may be able to learn whether enoxacin treatment can reverse learned helplessness after it has already been established. Enoxacin (and some of its fluoroquinolone analogs that also have effects on miRNA levels) is FDA-approved and widely used, making it an attractive reagent for study as way to modulate miRNAs in animals, and as a possible new therapeutic approach to human neuropsychiatric diseases.

## Conflict of Interest Statement

The University of Illinois at Chicago has filed a patent application for the use of enoxacin and related fluorquinolone compounds in the treatment of major depressive disorder and PTSD.

## Appendix

**Table A1:** **Confirmation experiment in a separate group of rats showing escape latency of individual rats when given no shock or inescapable shock and simultaneously treated with saline or enoxacin (10 or 25 mg/kg)**.

Shock or no shock^a^	Treatment	Escape latency (s)	Response to shock^b^
Sham (no shock)	Saline	9.684	
Sham (no shock)	Saline	5.324	
Sham (no shock)	Saline	9.216	
Sham (no shock)	Saline	3.496	
Sham (no shock)	Saline	3.732	
Sham (no shock)	Saline	4.152	
Sham (no shock)	Saline	3.19	
Sham (no shock)	Saline	6.36	
Shock	Enoxacin (10 mg/kg)	4.316	NLH
Shock	Enoxacin (10 mg/kg)	3.952	NLH
Shock	Enoxacin (10 mg/kg)	4.312	NLH
Shock	Enoxacin (10 mg/kg)	25.776	LH
Shock	Enoxacin (10 mg/kg)	9.656	NLH
Shock	Enoxacin (10 mg/kg)	9.26	NLH
Shock	Enoxacin (10 mg/kg)	26.968	LH
Shock	Enoxacin (10 mg/kg)	6.872	NLH
Shock	Enoxacin (10 mg/kg)	5.944	NLH
Shock	Enoxacin (25 mg/kg)	3.776	NLH
Shock	Enoxacin (25 mg/kg)	26.3	LH
Shock	Enoxacin (25 mg/kg)	7.088	NLH
Shock	Enoxacin (25 mg/kg)	2.512	NLH
Shock	Enoxacin (25 mg/kg)	4.444	NLH
Shock	Enoxacin (25 mg/kg)	3.244	NLH
Shock	Enoxacin (25 mg/kg)	29.476	LH
Shock	Enoxacin (25 mg/kg)	13.924	NLH
Shock	Enoxacin (25 mg/kg)	8.632	NLH

*^a^ Sham rats were given no shock on day 6 but were tested for escape latency on day 7*.

*^b^ For rats exposed to shock, escape latency <20 s = non-learned helpless (NLH); ≥20 s = learned helpless (LH)*.
